# Family support and satisfaction with mammography results communication

**DOI:** 10.1192/j.eurpsy.2026.11547

**Published:** 2026-07-31

**Authors:** I. Chaari, A. Samet, W. Abid, Y. Fourti, F. Charfeddine, L. Aribi, N. Messedi, J. Aloulou

**Affiliations:** Psychiatric department “B”, Hedi Chaker University Hospital, Sfax, Tunisia

## Abstract

**Introduction:**

The quality of mammography results communication is critical for patient-centered care. Family support may influence satisfaction with this process.

**Objectives:**

To explore whether family support predicts satisfaction with the communication of mammography results.

**Methods:**

We conducted an online survey of the general female population in Tunisia via a sponsored Facebook campaign (December 2023–February 2024). Participants rated family support (satisfactory vs unsatisfactory) and satisfaction with results communication. Logistic regression was applied.

**Results:**

A total of 278 women participated. The mean age was 46 years (SD 8.9, range 20–84). Most respondents were married (84.9%) and university educated (78.4%). Regarding socio-economic status, 66.5% reported middle income. Concerning communication, the majority received their mammography results face-to-face with a physician (81.3%), while smaller proportions were informed by non-physician healthcare staff (6.3%), by phone/email from a physician (3.0%) or by mail without interaction (0.4%). Other respondents didn’t specify the method. Satisfaction with communication was reported by 74% of women with satisfactory family support, compared to 42% with unsatisfactory support.

Women with satisfactory family support were significantly more likely to report satisfaction with results communication (74% vs 42%, χ² = 6.8, p = 0.009). Family support independently predicted satisfaction (OR = 2.20, 95% CI 1.25–3.86, p = 0.01).

**Conclusions:**

Among Tunisian women, family support is a strong determinant of satisfaction with results communication. Involving relatives in the process could enhance patient experience.

**Disclosure of Interest:**

None Declared

